# Unusual metallic state in superconducting A15-type La_4_H_23_

**DOI:** 10.1093/nsr/nwae149

**Published:** 2024-04-18

**Authors:** Jianning Guo, Dmitrii Semenok, Grigoriy Shutov, Di Zhou, Su Chen, Yulong Wang, Kexin Zhang, Xinyue Wu, Sven Luther, Toni Helm, Xiaoli Huang, Tian Cui

**Affiliations:** State Key Laboratory of Superhard Materials, College of Physics, Jilin University, Changchun 130012, China; Center for High Pressure Science and Technology Advanced Research (HPSTAR), Department of High-Temperature Superconductivity, Beijing 100193, China; Skolkovo Institute of Science and Technology, Skolkovo Innovation Center, Computational and Data Science and Engineering, Moscow 121205, Russia; Center for High Pressure Science and Technology Advanced Research (HPSTAR), Department of High-Temperature Superconductivity, Beijing 100193, China; State Key Laboratory of Superhard Materials, College of Physics, Jilin University, Changchun 130012, China; State Key Laboratory of Superhard Materials, College of Physics, Jilin University, Changchun 130012, China; State Key Laboratory of Superhard Materials, College of Physics, Jilin University, Changchun 130012, China; State Key Laboratory of Superhard Materials, College of Physics, Jilin University, Changchun 130012, China; Hochfeld-Magnetlabor Dresden (HLD-EMFL) and Würzburg-Dresden Cluster of Excellence, Helmholtz-Zentrum Dresden-Rossendorf (HZDR), Dresden 01328, Germany; Hochfeld-Magnetlabor Dresden (HLD-EMFL) and Würzburg-Dresden Cluster of Excellence, Helmholtz-Zentrum Dresden-Rossendorf (HZDR), Dresden 01328, Germany; State Key Laboratory of Superhard Materials, College of Physics, Jilin University, Changchun 130012, China; State Key Laboratory of Superhard Materials, College of Physics, Jilin University, Changchun 130012, China; School of Physical Science and Technology, Ningbo University, Ningbo 315211, China

**Keywords:** high-pressure, superconductivity, hydrides, negative magnetoresistance

## Abstract

Hydride superconductors continue to fascinate the communities of condensed matter physics and material scientists because they host the promising near room-temperature superconductivity. Current research has concentrated on the new hydride superconductors with the enhancement of the superconducting transition temperature (*T*_c_). The multiple extreme conditions (high pressure/temperature and magnetic field) will introduce new insights into hydride superconductors. The study of transport properties under very high magnetic fields facilitates the understanding of superconductivity in conventional hydride superconductors. In the present work, we report experimental evidence of an unusual metal state in a newly synthesized cubic A15-type La_4_H_23_ that exhibits superconductivity with a *T*_c_ reaching 105 K at 118 GPa. A large negative magnetoresistance is observed in strong pulsed magnetic fields in the non-superconducting state of this compound below 40 K. Moreover, we construct the full magnetic phase diagram of La_4_H_23_ up to 68 T at high pressure. The present work reveals anomalous electronic structural properties of A15-La_4_H_23_ under high magnetic fields, and therefore has great importance with regard to advancing the understanding of quantum transport behaviors in hydride superconductors.

## Teaser

An unusual metallic state with large negative magnetoresistance has been reported in A15-La4H23 superconductor under strong pulsed magnetic fields, expanding the understanding of quantum transport behaviors in hydride superconductors.

## INTRODUCTION

The search for high-temperature superconductors has been an important goal pursued tirelessly by researchers since the discovery of superconductivity (SC) in mercury [1]. However, until 2014, the critical temperature (*T*_c_) of conventional superconductors had never exceeded the McMillan limit (∼40 K) [2]. As one of the most effective methods of changing the structure of matter, pressure can lead to the appearance of unusual properties in materials that are unlikely to occur at ambient conditions, in particular, conventional high-temperature superconductivity (HTSC). The increase of *T*_c_ in Hg-containing cuprates to 164 K under high pressure motivated extensive research in this field [3]. On the basis of the chemical pre-compression idea, first proposed by Ashcroft [4], breakthrough investigations on conventional HTSC in compressed sulfur hydride H_3_S, with *T*_c_ above 200 K at 150 GPa, have been carried out both theoretically and experimentally since 2014 [5–9]. Soon after, the HTSC record was broken by lanthanum superhydride LaH_10_, with *T*_c_ = 250 K at 170 GPa [10,11]. The successful use of high pressure in the search for new superconductors provided a clear path to even room-temperature superconductivity and attracted considerable scientific interest. Over the past five years, such remarkable superconductors as H_3_S [6], LaH_10_ [10,11], ThH_10_ [12], YH_6_ [13], YH_9_ [14], CeH_9_, CeH_10_ [15], CaH_6_ [16,17] and (La, Ce)H_9-10_ [18,19] have been found.

To date, as one of the typical extreme conditions, high magnetic fields have brought about numerous unusual transport properties in conventional and unconventional superconductors. For example, the giant positive magnetoresistance (MR) in metal Ti [20] and WTe_2_ [21], as well as the negative MR, was observed in a series of cuprate superconductors [22,23]. However, the need to use ultrahigh pressure limits the study of transport properties. Only a few superhydrides [24–27] have been studied in pulsed magnetic fields up to now. Recently, a series of novel lanthanum hydrides were found using single-crystal X-ray diffraction (XRD) analysis, indicating their colorful superconducting phases at high pressure [28]. Hence, the La-H system is a perfect template for transport studies under high pressure and high magnetic fields.

In this work, we successfully synthesized a new representative of this class, cubic La_4_H_23_ with A15 structure, which is stable in the pressure range of 91–120 GPa, using laser heating of LaH_3-x_ with ammonia borane (AB, NH_3_BH_3_) above 1500 K. The electrical resistance exhibits a sharp drop by at least three orders of magnitude with the highest onset of *T*_c_ = 105 K. The A15 structural type was found for a series of intermetallic compounds with *Pm*$\bar{3}$*n* symmetry. These compounds exhibit outstanding low-temperature superconducting properties. Examples are vanadium silicide V_3_Si (*T*_c_ = 17 K) [29], Nb_3_Ge (*T*_c_ = 23.2 K) [30] and Nb_3_Sn (*T*_c_ = 18.3 K) [31]. Up to now, cubic La_4_H_23_ possesses the maximum *T_c_* recorded in the A15-structure class. Our theoretical calculations support the conclusions of the experiment and point to a strong electron–phonon interaction in the hydrogen sublattice of A15-La_4_H_23_. By the application of pulsed magnetic fields up to 68 T, we discovered a large negative MR region in the non-superconducting state of A15-type La_4_H_23_ below 40 K. The unusual metallic state in A15-La_4_H_23_ expands the understanding of quantum transport behaviors in hydride superconductors.

## RESULTS AND DISCUSSION

### High-temperature superconductivity of La_4_H_23_

**Figure 1.**
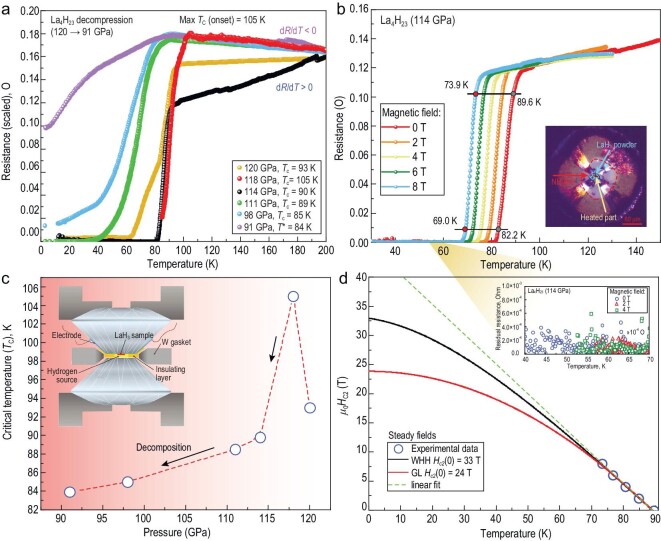
Pressure dependence of superconductivity in La_4_H_23_. (a) Temperature dependence of the electrical resistance of the sample during decompression of DAC-H1 from 120 GPa to 91 GPa. *T*_c_ corresponds to the transition onset point. (b) Magnetic field dependence of the electrical resistance of the sample at 114 GPa. Inset: photograph of the sample loaded in the DAC's chamber and four Mo electrodes after laser heating at 114 GPa. (c) Pressure dependence of the critical temperature of La_4_H_23_. Inset: schematic diagram of the electrical DAC with the four-electrode van der Pauw scheme. (d) Upper critical magnetic field *B_С_*_2_*(T)* of La_4_H_23_ at 114 GPa obtained by extrapolation of the experimental data (steady magnetic fields) using the GL [42], the WHH [43,44] and linear models. Inset: residual resistance of the sample below the superconducting (SC) transition.

For the experimental transport measurements, we prepared two diamond anvil cells, DAC-H1 and H2. The scheme of the electrical DAC-H1 prepared for the transport measurements is shown in Fig. 1b and c. Laser heating of the LaH_3-x_/NH_3_BH_3_ (AB) sample to a temperature above 1500 K was performed at 123 GPa. Afterwards, the pressure was reduced to 120 GPa. Cryogenic measurements of the sample showed immediately the appearance of a drop in the electrical resistance from 0.12 Ω to 10^−4^ Ω at 93 K (onset) corresponding to the manifestation of superconductivity in the sample (Fig. 1a). After the second laser heating, a sharp superconducting transition was observed at 90 K at 114 GPa. The third laser heating led to an increase in pressure to 118 GPa and the critical temperature *T*_c_ also increased to 105 K. This value is very close to the values of cerium superhydrides CeH_9_ and CeH_10_ in the same pressure range. However, in La_4_H_23_ the same critical parameters are achieved with a significantly smaller amount of hydrogen (<6 atoms per La, see below). To further investigate the dependence of *T*_c_ on pressure, we decompressed the DAC to 91 GPa (Fig. 1a). We found that *T*_c_ (P) decreased monotonically during the decompression runs with its maximum of 105 K at 118 GPa (Fig. 1c).

The overall normal-state resistance follows the temperature almost linearly (see Fig. 1a). Remarkably, the slope of quasi-T-linear resistance, *dR/dT*, changes its sign during decompression (Fig. S7). This phenomenon has already been observed in the sulfur hydrides H_2_S and H_3_S [6], phosphorus hydride PH_3_ [32], cerium hydride CeH_10_ [27] and ternary hydride (La, Ce)H_9_ [18]. As we know from previously reported study of SnH_4_ [33], the change in the sign of *dR/dT* is incompatible with the view of La_4_H_23_ as a normal Fermi-liquid metal whose electrical resistance is due to electron-phonon scattering. Indeed, the influence of electron–phonon interaction on the transport properties of metals can be described using the Eliashberg transport spectral function α^2^*F*_tr_(ω), which, in general, differs little from the Eliashberg function for electron–phonon interaction α^2^*F*(ω) [34]. In the first approximation of the variational solution of the Boltzmann equations for electron transport [35], the dependence of electrical resistivity (*ρ*) on temperature (*T*) is linear:


(1) \begin{eqnarray*}
\rho\! \left( T \right) = T \displaystyle\frac{{\pi {{V}_{{cell}}}{{k}_B}}}{{{{N}_F}\!\left\langle {v_x^2} \right\rangle }}\mathop \int \nolimits_0^\infty \displaystyle\frac{{d\omega }}{\omega } \displaystyle\frac{{{{x}^2}}}{{s{{h}^2}\!\left( x \right)}}{{{\mathrm{\alpha }}}^2}{{F}_{tr}}\!\left( {\mathrm{\omega }} \right),
\end{eqnarray*}

where *x* = $\hbar \omega /{{k}_B}T$, *V*_cell_ is the unit cell volume, *N*_F_-is the electron density of states at the Fermi level and $\langle {v_x^2} \rangle $ is the band-averaged electron Fermi velocity. All of the above parameters are positive in all known 3D materials, which leads to a positive *dR/dT* for the vast majority of metals and alloys. Very rare exceptions are complex alloys with disorder effects at low temperatures, such as manganin and constantan [36–38]. At high temperatures, the term $\frac{{{{x}^2}}}{{s{{h}^2}( x )}}$ approaches to 1, and we obtain the simple expression:


(2) \begin{eqnarray*}
\rho\! \left( T \right) = {{\lambda }_{tr}}\displaystyle\frac{{\pi {{V}_{{cell}}}{{k}_B}}}{{{{N}_F}\!\left\langle {v_x^2} \right\rangle }}T,
\end{eqnarray*}

where *λ*_tr_ is the transport electron–phonon coupling (EPC) parameter [34]. Consequently, if *dR/dT* < 0, the transport EPC parameter must be negative (λ_tr_ < 0), and the usual EPC strength (λ) will also be negative or about zero, which is incompatible with the concept of conventional superconductivity in La_4_H_23_. A similar behavior has been independently found by Cross *et al*. [39] and, therefore, is reproducible.

The properties of polyhydrides in the non-superconducting state on the verge of their dynamic stability [40] should be described in the framework of a non-Fermi-liquid model similar to the models developed for describing the pseudogap, strange-metal phase and metal-to-insulator transitions in cuprates [41]. As we will see in the next paragraph, A15-La_4_H_23_ exhibits a strong negative MR at high fields that is related to its unusual metallic state. As can be seen from Fig. 1с, a further decrease in pressure of DAC-H1 leads to broadening of the superconducting transitions up to 98 GPa. The decomposition of La_4_H_23_ could be triggered at 91 GPa, indicated by the disappearance of the superconducting state.

To further confirm the superconductivity, we measured the electrical resistance of the sample in DAC-H1 in steady magnetic fields ranging from 0 to 8 T. Figure 1b shows the temperature dependence of the electrical resistance of A15-La_4_H_23_ in steady magnetic fields at 114 GPa. The sample demonstrates the absence (and even negative value) of a broadening of superconducting transitions in a magnetic field, as previously observed for yttrium (YH_6_) [45] and lanthanum-yttrium ((La, Y)H_10_) hydrides [46]. *T*_c_ shifts linearly to lower temperatures with increasing magnetic fields, as expected for superconductors. To estimate the upper critical magnetic field *μ_0_H_C_*_2_(0), we applied the Ginzburg-Landau (GL) model [42], and the Werthamer-Helfand-Hohenberg (WHH) model [43], simplified by Baumgartner [44] (Figure 1d). The extrapolation of *μ_0_H_C_*_2_(0) to zero temperature yields 24 T and 33 T for the GL and WHH models, respectively. The coherence length can be estimated by *μ_0_H_C_*_2_ = *Φ*_0_/(2π*ξ*^2^) [47]. The *ξ_WHH_*(0) and *ξ_GL_*(0) are equal to 3.16 nm and 3.72 nm, respectively. These values are much higher than the ones for *fcc*-LaH_10_ [47].

Interestingly, the superconducting properties of La_4_H_23_ are found to be more pronounced than those of the recently studied A15-Lu_4_H_23_ (max *T*_c_ = 71 K [48]). This indicates that isostructural lutetium compounds, such as proposed LuH_10_, LuH_9_ and LuH_6_, will have lower critical temperatures than the corresponding lanthanum polyhydrides, in contradiction with earlier theoretical predictions [49]. Moreover, due to the smaller atomic radius of Lu, we would have to apply much higher pressures to stabilize the corresponding lutetium hydrides (e.g. LuH_10_), making them less convenient to study than LaH_x_.

### The X-ray diffraction data of La_4_H_23_

The structural type A15 (*Pm*$\bar{3}$*n*) was found for the first time during studies of the formation of europium hydrides [50] above 100 GPa. Later on, polyhydrides with the same structure were found in the Ba-H system [51], among La [28] and Lu [48] polyhydrides, and in the Y-H system [52], forming a fairly large family of superhydrides. As we described in the previous section, A15-La_4_H_23_ has the highest *T*_c_ of all superconductors with an A15 structure known to date. In the following, we discuss the results of XRD analysis of this compound.

**Figure 2.**
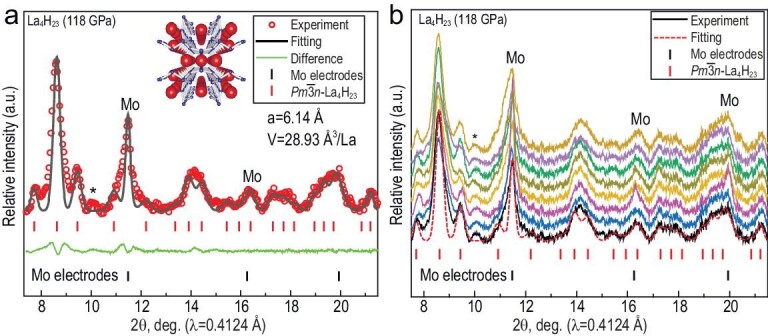
X-ray diffraction (XRD) analysis of La_4_H_23_. (a) XRD pattern and Le Bail refinement of the unit cell parameters of La_4_H_23_ phase at 118 GPa. Experimental XRD data and the Le Bail fit are represented by red hollow circles and black lines, respectively. The XRD pattern contains spurious signals from molybdenum (Mo) electrodes. Inset: crystal structure of La_4_H_23_ in a perspective projection. (b) XRD patterns measured across the sample in steps of 5 μm. The sample is very homogeneous.

Considering the large number of superconducting phases in the La-H system at megabar pressures, we combined the experimental powder XRD data and the computational crystal structure search [28]. The *in-situ* synchrotron XRD patterns, shown in Fig. 2a, indicate that the La sublattice of the DAC-H1 sample possesses a cubic *Pm*$\bar{3}$*n* symmetry at 118 GPa. The volume of the unit cell is 28.9 Å^3^/La and the unit cell parameter is a = 6.14 Å (Z = 8). This is in close agreement with the theoretical results in 118 GPa (≈ 28 Å^3^/La, see Fig. S13). We also compared the obtained unit-cell volume of $Pm\bar{3}n$-La_4_H_23_ with the reported one [28], which is 27.98 Å^3^/La at 150 GPa. This is slightly different due to a higher pressure in the Laniel *et al*. experiment. The hydrogen content can be estimated by the difference between the atomic volumes of La and H atoms [53,54]. According to ref. [55], the volume of a hydrogen atom in pure compressed hydrogen at 118 GPa is 2.20 Å^3^/H, whereas the La has a volume of 15.7 Å^3^/La [53]. Combining these data, we conclude that the La : H composition in our compound is close to 1 : 6. Figure 2b shows a series of XRD patterns measured at several positions across the sample in steps of 5 μm. All XRD images are almost identical at all locations containing the signals from the Mo electrodes and $Pm\bar{3}n$-La_4_H_23_. This confirms the uniform distribution of hydrogen and selective formation of only one phase in the process of synthesis. Computer modeling of this structure shows that in the hydrogen cage of La_4_H_23_, the shortest H-H distance is ∼1.3 Å at 118 GPa, which is in the range of 1.0–1.5 Å, typical for hydride superconductors. This bond length is much longer compared to pure H_2_: *d*_HH_ ≈ 1.1 Å at 115 GPa [10].

### Pulsed magnetic field experiments of La_4_H_23_

**Figure 3.**
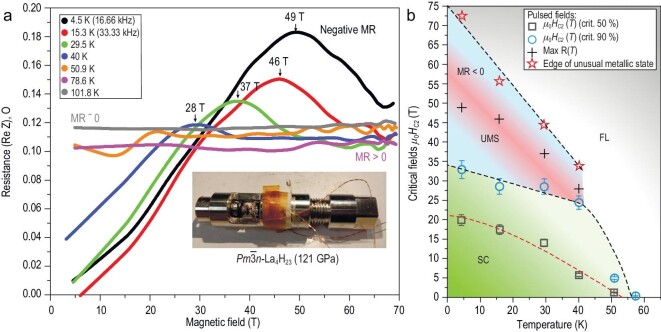
Transport properties in pulsed magnetic fields. (a) Magnetic field dependence of the electrical resistance *R(H)* of the sample in DAC-H2 measured in the AC mode at frequencies of 16.66 kHz (4.5 K) and 33.33 kHz (most cases). For ease of analysis, the original data were smoothed using a Fourier filter (Origin lab). Raw data can be found in the Supplementary Data. Inset: the DAC used in pulsed field experiments. (b) Magnetic phase diagram of La_4_H_23_ at 121 GPa. ‘SC’ marks the superconducting region. The unusual metallic phase corresponds to the red (max *R(T)* line) and top blue regions where MR < 0. ‘FL’ denotes the Fermi-liquid metal behavior of the sample with MR > 0 and positive d*R/*d*T* > 0.

The unusual transport properties of La_4_H_23_ motivated us to conduct studies in even higher magnetic fields up to 68 T. For this, a second assembly DAC-H2 (inset of Fig. 3a) was prepared using the same LaH_3-x_/AB starting material at a pressure of 121 GPa. The DAC-H2 is suitable to fit into a pulse-magnet set-up. Before the pulsed-field experiment, DAC-H2 was tested in steady magnetic fields up to 8T (Figs S8–S9). DAC-H2 exhibits superconductivity below 84 K (onset) with an extrapolated *μ_0_H_C_*_2_(0).

A pulsed magnetic field provided us with access to a region of pronounced negative MR at temperatures below 40 K and at fields *μ_0_H* > 25 T (see Fig. 3a). By tracing the inflection points, where the MR changes its sign back to positive, we were able to create a schematic phase diagram, shown in Fig. 3b. The broad region with negative MR emerges right above the superconducting phase, which is called the unusual metallic state. The electrical resistance exhibits a maximum peak associated with the strange metal phase, and the peak value increases as the temperature decreases. *R_max_* reaches a maximum of 0.185 Ω at 4.5 K and decreases with the increasing of the temperature to *R*_n_ = 0.115 Ω at 50.9 K. A similar phenomenon was observed in CeH_10_ [27] superhydride and many cuprates [22,56,57], which is possibly attributed to the pseudogap phase. This phenomenon has been associated with strong correlation effects. In the Hubbard-Holstein model, collective charge fluctuations mediate the temperature-dependent scattering of quasi-particles [58]. The pseudogap opening leads to a decrease in the electronic density state at the Fermi level and the number of charge carriers. Along with the increase in applied magnetic fields, the pseudogap is suppressed gradually, and the *R(H)* slope is negative. The observed large negative MR in La_4_H_23_ closely resembles the magnetoresistance features which associated with the pseudogap in some cuprates, such as Nd_2-x_Ce_x_CuO_4_ [57], Bi_2_Sr_2_CaCu_2_O_8+y_ [22] and Bi_1.6_Pb_0.4_Sr_2_CaCu_1.96_Fe_0.04_O_8+δ_ [56]. In all of the above cases, the field dependence of the electrical resistance of samples is very similar to the behavior in La_4_H_23_.

It is interesting to note that above the superconducting transition, at 101.8 K, the MR remains zero over the full accessible field range: *MR* = d*ρ*/d*B* = 0. For normal metals, the MR is positive and *MR = (R − R_0_)/R_0_*  $\propto \ $*μ^2^H^2^*, where *R* is the resistance in external magnetic fields (*B*), *R_0_* is the normal state resistance before superconducting transition, and *μ* is the charge carrier mobility (Fig. S10). This shows that there are several contributions of opposite signs in the MR of La_4_H_23_, which can cancel each other out in some temperature ranges.

We were also able to entirely suppress superconductivity at *μ_0_H_C_*_2_(0) ≈ 32 T in the La_4_H_23_ and complete the magnetic phase diagram for this compound (Fig. 3b). In general, the behavior of *μ_0_H_C_*_2_*(T)* is in agreement with the WHH model traditionally used for Bardeen-Cooper-Schrieffer superconductors. Moreover, in the region of *μ*_0_*H* ≫ *μ*_0_*H*_C2_, the positive MR and d*R/*d*T* is consistent with what is expected from Fermi-liquid theory.

To further analyze the metallic state, we performed fits to the *R*–*T* curves in the non-superconducting range based on the equation:


(3) \begin{eqnarray*}
R\!\left( T \right) = M + A{{T}^n},\end{eqnarray*}

where *M* is the residual resistance, and *A* and *n* are the coefficients of *T*-square resistance and the power exponent of temperature, respectively (Fig. S11). According to the present data quality, the temperature dependence of electrical resistance of the ordinary metallic state of La_4_H_23_ can only be described qualitatively within the Fermi-liquid model.

The effect of negative MR in hydrides may depend on sample preparation, concentration of defects and the average crystallite size. The latter (*d_cryst_*) must be greater than the Larmor radius:


(4) \begin{eqnarray*}
{{d}_{{cryst}}} \approx {{l}_e} \gg {{r}_g} = \frac{{m_e^*{{v}_F}}}{{eB}},\end{eqnarray*}

where *l_e_* is the electron mean-free path, *v_F_* can be estimated as ∼3 × 10^5^ m/s [59] and m_e_* is the effective electron mass. Otherwise (${{l}_e} \ll {{r}_g}$) the MR will be near zero due to dominant scattering at grain boundaries. If we take m_e_* = m_e_ and magnetic field *μ_0_H* = 100 T, then *r_g_* ∼ 19 nm. This value is comparable to the lattice period of superhydrides. However, in a field of 10 T, *r_g_* ∼ 190 nm and in many superhydride samples, the crystallite size and the average distance between defects will already be shorter. In this case, scattering at defects and grain boundaries will be the dominant process, and the influence of *μ_0_H* will be negligible.

Furthermore, we should consider a positive quadratic contribution to MR $\propto \mu _e^2\mu _0^2{{H}^2}$ (here *μ_e_* is the electron mobility), observed due to the distortion of electron trajectories in an external magnetic field. Regardless of the dependence of the Fermi energy *E_F_*, Fermi velocity *v_F_*, density of state *N_F_* and m_e_* on pressure, temperature and hydrogen content, this effect will be observed. It may therefore compensate for the negative MR. We believe that its origin is associated with the preformation of Cooper pairs and other effects responsible for the pseudogap phase and strange metal behavior of superconductors. Consequently, in some cases the negative MR can be overlaid by these additional contributions and, hence, may be hidden from experiments.

### Theoretical analysis

**Figure 4.**
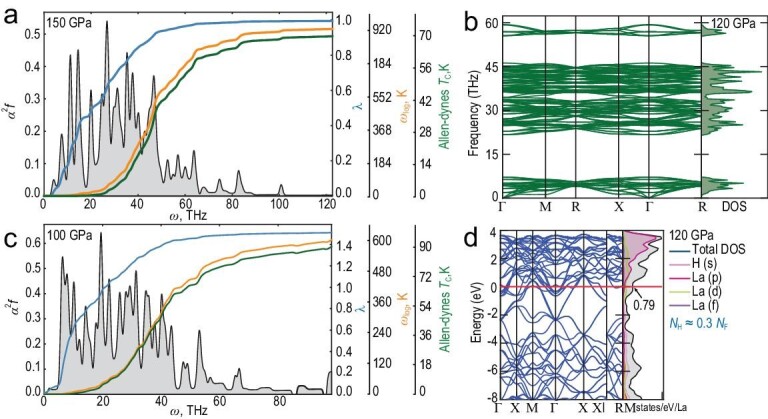
Results of theoretical calculations of the electronic, phonon and superconducting properties of La_4_H_23_ at 100, 120 and 150 GPa. (a and c) Eliashberg functions of La_4_H_23_ calculated without considering symmetry at 150 and 100 GPa, respectively. We used a *k*-mesh of 8 × 8 × 8 and a *q*-mesh of 2 × 2 × 2. The results were also verified on a *k*-mesh 12 × 12 × 12 and a *q*-mesh 3 × 3 × 3. Pictures were prepared using a python script available on GitHub [60]. (b) Phonon band structure and density of states of La_4_H_23_ at 120 GPa calculated in the harmonic approximation. The compound was found to be dynamically stable at this pressure. The compound has weak imaginary phonon modes at 100 GPa, that disappear after considering the anharmonic effects (Fig. S12). (d) Electron band structure and density of states projected on H and La atoms at 120 GPa. The contribution of hydrogen is ≈30% of the total density of states.

To confirm the experimental observations and find the most probable hydrogen sublattice in the synthesized lanthanum hydride, we performed a series of thermodynamic calculations of the enthalpies of formation for various La polyhydrides at 100, 120, 150 and 200 GPa, and temperatures from 0 to 2000 K, considering the zero-point energy (ZPE) contribution in the harmonic approximation. The corresponding convex hulls are shown in Figs S1–S6, and the enthalpies of formation can be seen in Tables S1–S3. As can be seen, $Pm\bar{3}n$-La_4_H_23_ is close to the stability region and ∼20–30 meV/atom above the convex hull at 100–150 GPa. This phase is dynamically stable at 120 GPa already in the harmonic approximation (Fig. 4b), although further pressure reduction to 100 GPa destabilizes it, in agreement with the experiment (Fig. 1a). Increasing the temperature has a little effect on its relative stability (Figs S4–S6), whereas the stability of the neighboring highly symmetric phases $Im\bar{3}m$-LaH_6_ and $Fm\bar{3}m$-LaH_10_ is strongly affected. We found that $Im\bar{3}m$-LaH_6_ is dynamically unstable in the harmonic approximation at 100–120 GPa and can undergo a distortion $Im\bar{3}m$ → *Cmme* → *C*2/*m* at low pressure, which stabilizes La_4_H_23_. This is also supported by the known experimental fact of distortion of $Fm\bar{3}m$-LaH_10_ at pressures below 140 GPa [24]. Thus, the convex hull deformation at low pressures, caused by a much faster destabilization of the $Im\bar{3}m$*-*LaH_6_, may lead to possible experimental observation of $Pm\bar{3}n$-La_4_H_23_. Indeed, all lanthanum hydrides discussed above were synthesized in recent experiments [28].

Superconducting properties of $Pm\bar{3}n$-La_4_H_23_ have been investigated theoretically using the norm-conserving Hartwigsen-Goedecker-Hutter (HGH) pseudopotentials with the Perdew-Zunger (PZ) functional (Fig. S14). These pseudopotentials give results that are in close agreement with the experimental data, therefore this computational approach will be discussed below (Table 1 and Fig. 4a and c). We find that La_4_H_23_ exhibits moderate superconducting properties at 100 GPa, and the electron–phonon interaction strength reaches λ ≈ 1.5. The critical temperature of superconductivity decreases with increasing pressure due to a decrease in λ. The calculated *T*_c_ of La_4_H_23_ reaches 92–95 K at 100 GPa in the harmonic approximation, in agreement with the experiment. The relatively low *T*_c_ of this superhydride correlates with the low contribution of the hydrogen sublattice to the density of electronic states (Fig. 4d), which is equal to ∼30% of the total density of electronic states at the Fermi level (*N*_F_ = 0.79 states/eV/La). There is a significant contribution of *d* and *f* electrons to the density of electronic states at the Fermi level. The expected [61] upper critical magnetic field *μ_0_H_C_*_2_(0) = 29–51 T is also in reasonable agreement with the experiment (Fig. 3b).

**Table 1.** Parameters of the superconducting state of $Pm\bar{3}n$-La_4_H_23_ calculated at 100 and 150 GPa in the harmonic approximation using HGH PZ pseudopotentials. ‘McM’ stands for the McMillan formula [2], ‘A-D’ stands for the Allen-Dynes model [63] and ‘E’ corresponds to the solution of the isotropic Eliashberg equations [64] (μ* = 0.1 in all cases).

Pressure	100 GPa	150 GPa
λ	1.49	1.0
ω_log_, K	650	892
ω_2_, K	1121	1383
*T*_c_ (McM)	73.5	61.4
*T*_c_ (A-D)	91.7	68.0
*T*_c_ (E)	94.7	70.4
*N*_F_, states/spin/Ry/Å^3^	0.196	0.182
2Δ*/k_B_T*_c_	4.69	4.04
Expected *μ_0_H_C2_(0), T*	51	29
2Δ, meV	19.1	12.2

All lanthanide hydrides have a pronounced contribution of *d*- and *f*-electrons to the density of electronic states and can be classified as strongly correlated systems. For density functional theory (DFT) calculations of lanthanide compounds, the use of the DFT + U formalism is very popular [50,62], which considers strong correlations in the electronic subsystem. In this regard, the discovery of the strange-metal state with similarities to a pseudogap phase in La_4_H_23_ and cerium hydride CeH_10_ [27] is not completely unexpected.

## CONCLUSIONS

We have discovered a new lanthanum superhydride, cubic A15-type La_4_H_23_, with lower stabilization pressure than the reported *fcc*-LaH_10_. The maximum *T*_c_ of A15-type La_4_H_23_ is 105 K at 118 GPa, evidenced by the sharp decrease of electrical resistance and the shift of the resistive transition in applied magnetic fields. This new lanthanum superhydride could be stabilized down to 90 GPa; hence, it belongs to the few hydride superconductors that are stable below 100 GPa with *T*_c_ above 100 K. At high magnetic fields, the investigated La_4_H_23_ exhibits pronounced properties of an unusual metallic state: a large *H*-linear negative MR, a sign reversal of the temperature coefficient of electrical resistance (d*R/*d*T*) below 40 K, and a quasi-linear *R*(*T*) in the non-superconducting state. Our experimental findings expand and advance the understanding of physics of hydride superconductors.

## EXPERIMENTAL METHODS

### Sample synthesis

Two cells (DAC-H1 and DAC-H2) were prepared for transport measurements. DAC-H2 was used for the pulsed-field magnetotransport measurements. We used powder LaH_3-x_ (x ≈ 0.1–0.2) with metallic conductivity, which was preliminarily synthesized from La powder and hydrogen under pressure, and ammonia borane (AB, NH_3_BH_3_) as the hydrogen source and pressure transmitting medium, considering the high-temperature decomposition reaction: NH_3_BH_3_→3H_2_ + *c*-BN [15]. The *in-situ* synchrotron XRD patterns were obtained at BL10XU of the Spring-8 facility (λ = 0.4124 μm). The CeO_2_ was used as a calibrant. The Dioptas software was used to analyze the experimental XRD patterns [65].

### High pressure set-up

The DACs were made from NiCrAl (40HNU) alloy for high-pressure experiments. The diamond anvils had a culet with a diameter of 60 μm, beveled at 8° to a diameter of 250 μm. For transport measurements, MgO/epoxy was selected as the insulating layer placed between the tungsten gasket and platinum-foil leads. Molybdenum electrodes were sputtered on the surface of the diamond anvils to connect the sample and Pt leads. The pressure was determined by the diamond Raman shift [66]. Further experimental details are provided in the Supplementary Data.

### Four-probe electrical transport measurements

The four-probe scheme is used in the electrical transport measurements. Four molybdenum electrodes were sputtered onto the surface of diamond anvils and connected to external cooper wires using 3 μm platinum foils. We carried out the resistance measurements in a multifunctional measurement system (1.5–300 K, JANIS Research Company Inc, 0–9 T, Cryomagnetics Inc.).

### Pulsed-field magnetotransport measurements

MR measurements in pulsed magnetic fields were carried out in a resistive pulse magnet with 24 mm bore and a maximum field of 70 T (pulse duration of 150 ms) at the Dresden High Magnetic Field Laboratory (HLD-EMFL). We connected twisted cooper wires via strands of Litz wire to the Mo leads on the DAC. In order to minimize induced voltages from open-loop areas, we fixed Litz wires, with the help of silver paint, as closely together as possible. All twisted pairs were immobilized using GE7031 varnish. In order to achieve excellent control of the DACs temperature between 4.5 and 78 K, a helium-bath cryostat was used. A 100-cm-long NiCr wire with a resistance of ∼150 Ohms, wrapped around the diamond chamber, was used as a heater. Cernox thermometers were directly attached to the DAC's body (60 mm long and 12 mm in diameter) for measurements of the temperature. We applied a four-probe AC method with an excitation current of 1–2 mA and high frequencies between 16 and 33 kHz. Note: we made sure to stay well below the critical current of the sample. For 10 mA the superconducting transition started to get significantly suppressed. A digital lock-in and filter were applied to the raw data afterwards. The voltage drop across the sample was amplified by an instrumentation amplifier and detected by a lock-in amplifier. In general, we used the same methodology as in previous studies of (La, Nd)H_10_ [25], SnH_4_ [33] and CeH_9-10_ [27].

## COMPUTATIONAL DETAILS

The evolutionary algorithm USPEX [67–69] was used to predict thermodynamically stable La-H phases. To investigate the La–H system, we performed both fixed- and variable-composition searches at 100, 120, 150 and 200 GPa. The number of generations was 100. We calculated the convex hulls in the temperature range 0–2000 K, using free energies computed by Phonopy [70]. Metastable structures with the energy ≤30 meV/atom above the hull are also presented on the convex hulls.

Structure relaxations and energy calculations were performed using the VASP code [71–73] within DFT [74,75], implementing the Perdew–Burke–Ernzerhof (PBE) exchange–correlation functional [76] and the projector-augmented wave (PAW) method [77,78]. The kinetic energy cut-off was set at 600 eV. Γ-centered k-point meshes with a resolution of 2π × 0.05 Å^−1^ were used for sampling the Brillouin zone. The phonon band structure and density of states were computed using the Phonopy [70] package, implementing the finite displacement method. 2 × 2 × 2 supercells were generated. The energy cut-off and k-spacing parameters for the VASP calculations were set at 500 eV and 2π × 0.1 Å^−1^, respectively. The Sumo package [79] was used to visualize the phonon density of states and band structure. The k-points for the phonon band structures were chosen using Hinuma's recommendation [80]. The Phonopy package was also used to calculate ZPE corrections and thermal properties, such as entropy and free energy. To calculate phonon frequencies and EPC coefficients, we used the Quantum Espresso (QE) package [81], utilizing density functional perturbation theory (DFPT) [82], plane-wave PZ HGH pseudopotentials and the tetrahedron method [83,84]. In general, we used the same methodology as in the study of the La-Mg-H system [85].

## Supplementary Material

nwae149_Supplemental_File
