# Supplementary material for: Unusual metallic state in superconducting A15-type La4H23
Source: Natl Sci Rev. 2024 Apr 18;11(12):nwae149. doi: 10.1093/nsr/nwae149 (PMC11562835; doi:10.1093/nsr/nwae149)
Supplement: nwae149_Supplemental_File [file nwae149_supplemental_file.docx]

SUPPORTING INFORMATION

**Unusual metallic state in superconducting A15-type La_4_H_23_**

Jianning Guo ^1, †^, Dmitrii Semenok^2, †,^ *, Grigoriy Shutov ^3, †^, Di Zhou^2^, Su Chen^1^, Yulong Wang^1^, Kexin Zhang^1^, Xinyue Wu^1^, Toni Helm^4^, Sven Luther^4^, Xiaoli Huang^1,^ *, and Tian Cui^1,5,^ *

*^1^ State Key Laboratory of Superhard Materials, College of Physics, Jilin University, Changchun 130012, China*

*^2^ Center for High Pressure Science and Technology Advanced Research (HPSTAR), Beijing*

*^3^ Skolkovo Institute of Science and Technology, Skolkovo Innovation Center, 3 Nobel Street, Moscow 143026, Russia*

*^4^ Hochfeld-Magnetlabor Dresden (HLD-EMFL) and Würzburg-Dresden Cluster of Excellence, Helmholtz-Zentrum Dresden-Rossendorf (HZDR), Dresden 01328, Germany*

*^5^ School of Physical Science and Technology, Ningbo University, Ningbo 315211, China*

^†^These authors contributed equally to this work

^*^Corresponding authors, emails: [dmitrii.semenok@hpstar.ac.cn](mailto:dmitrii.semenok@hpstar.ac.cn) (D. Semenok)

[huangxiaoli@jlu.edu.cn](mailto:huangxiaoli@jlu.edu.cn) (X. Huang)

[cuitian@nbu.edu.cn](mailto:cuitian@nbu.edu.cn) (T. Cui)

**Content**

1. Thermodynamic calculations …………………………………………………………………………S2

2. Electron, phonon, elastic and superconducting properties of La_4_H_23_…………………………………S7

**1. Thermodynamic calculations**

**Figure S1.** Convex hull of the La-H system calculated at 100 GPa and 0 K in harmonic approximation. Only those phases that lie less than 30 meV/atom from the convex hull are shown. Zero-point energy (ZPE) was included in the calculations.

**Figure S2.** Convex hull of the La-H system calculated at 120 GPa and 0 K in harmonic approximation. Only those phases that lie less than 30 meV/atom from the convex hull are shown. Zero-point energy (ZPE) was included in the calculations.

**Figure S3.** Convex hull of the La-H system calculated at 150 GPa and 0 K in harmonic approximation. Only those phases that lie less than 30 meV/atom from the convex hull are shown. Zero-point energy (ZPE) was included in the calculations.


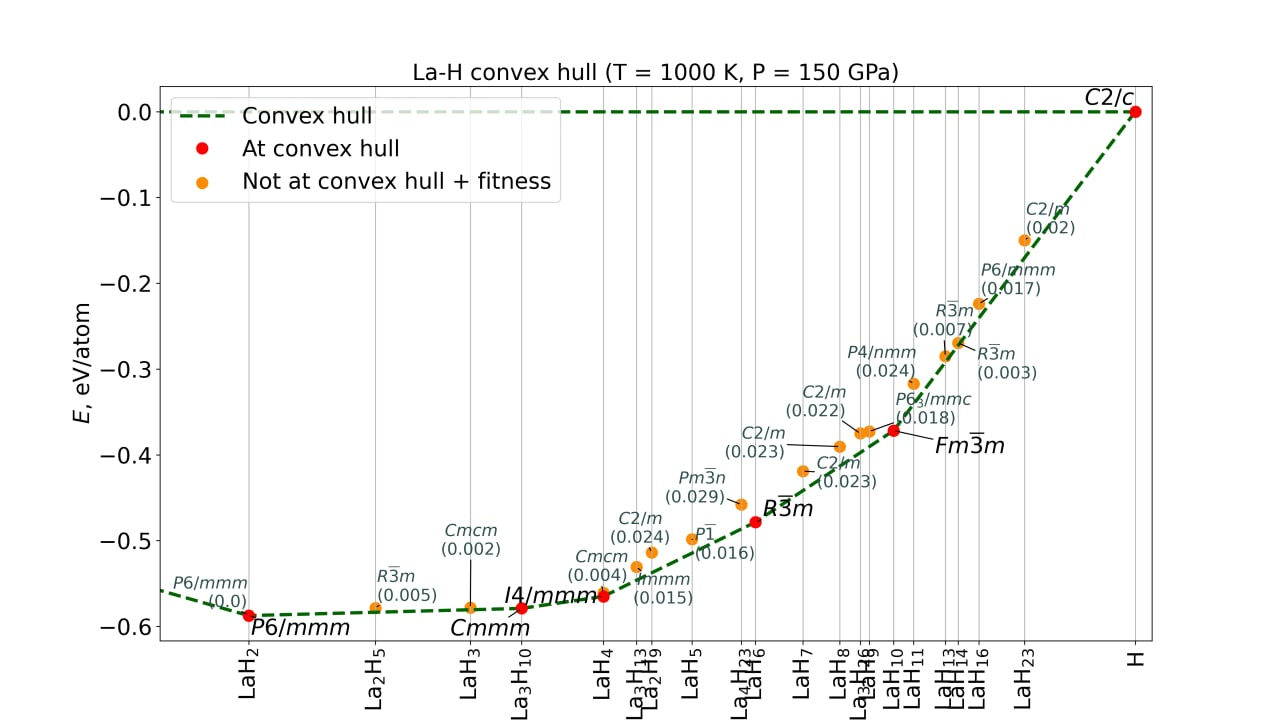


**Figure S4.** Convex hull of the La-H system calculated at 150 GPa and 1000 K in harmonic approximation. Only those phases that lie less than 30 meV/atom from the convex hull are shown. Zero-point energy (ZPE) was included in the calculations. The numbers in parentheses correspond to the distance of the connections from the convex hull in eV/atom.


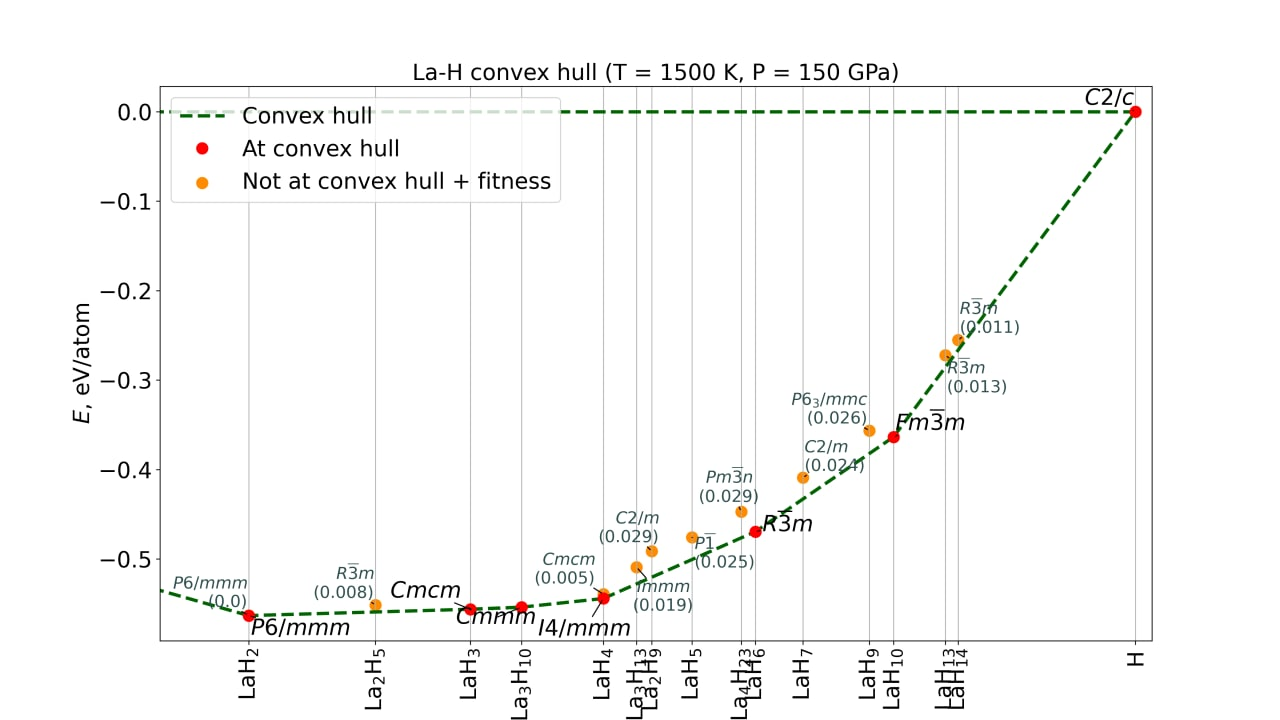


**Figure S5.** Convex hull of the La-H system calculated at 150 GPa and 1500 K in harmonic approximation. Only those phases that lie less than 30 meV/atom from the convex hull are shown. Zero-point energy (ZPE) was included in the calculations. The numbers in parentheses correspond to the distance of the connections from the convex hull in eV/atom.


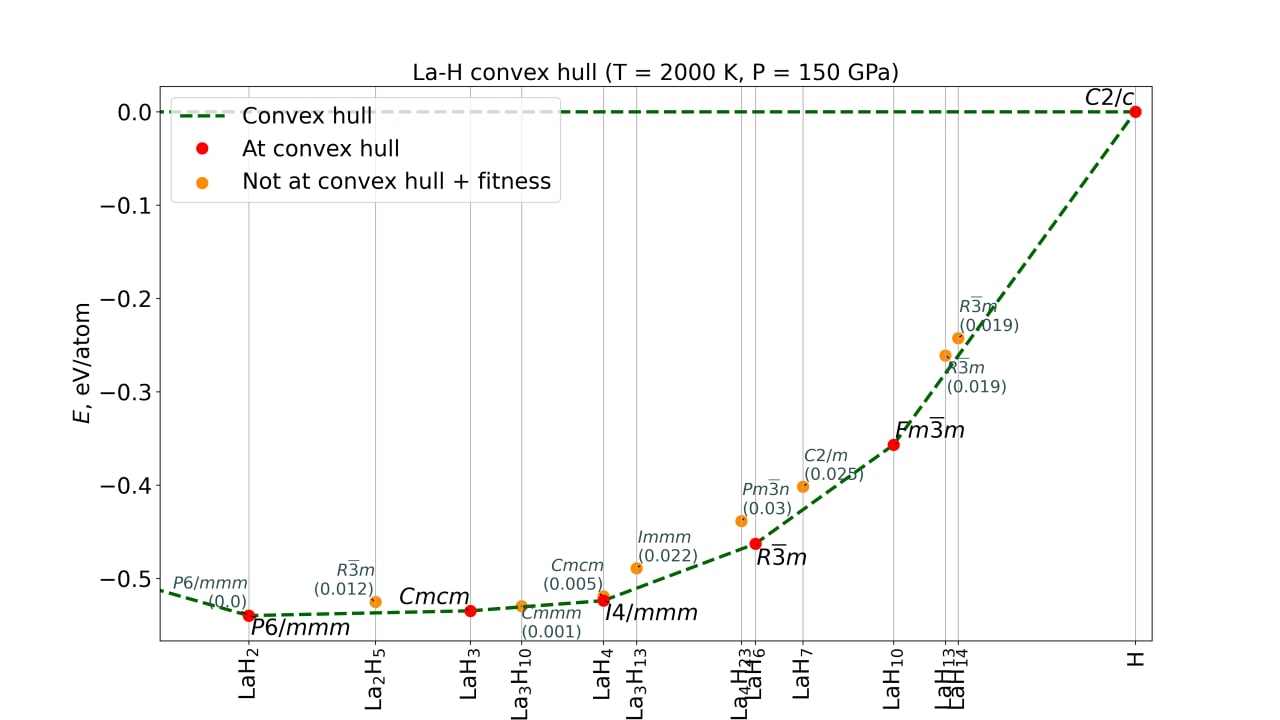


**Figure S6.** Convex hull of the La-H system calculated at 150 GPa and 2000 K in harmonic approximation. Only those phases that lie less than 30 meV/atom from the convex hull are shown. Zero-point energy (ZPE) was included in the calculations. The numbers in parentheses correspond to the distance of the connections from the convex hull in eV/atom.

**Table S1.** Enthalpies of formation of various lanthanum hydrides (in eV/atom) at 100 GPa and 0 K (Figure S1). ZPE stands for the zero-point energy. X, Y are coordinates of lanthanum hydrides on the two-dimensional convex hull diagram. Fitness is the distance from the convex hull line.

| **Space Group** | **Formula** | **X** | **E** | **ZPE** | **E+ZPE** | **Y** | **ZPE Y** | **Fitness without ZPE** | **ZPE fitness** |
| --- | --- | --- | --- | --- | --- | --- | --- | --- | --- |
| *Fm-3m* | LaH_10_ | 0,90900 | -0,62980 | 0,15270 | -0,47710 | -0,26900 | -0,35680 | 0,03340 | 0,00 |
| *C2/m* | La_2_H_9_ | 0,81800 | -0,04020 | 0,19930 | 0,15910 | -0,52800 | -0,54790 | 0,01110 | 0,01730 |
| *P4/nmm* | LaH_11_ | 0,91700 | -0,72460 | 0,22610 | -0,49850 | -0,29300 | -0,30920 | 0,00 | 0,01620 |
| *C2/m* | LaH_23_ | 0,95800 | -0,96460 | 0,24850 | -0,71610 | -0,14400 | -0,14760 | 0,00280 | 0,01710 |
| *R-3m* | LaH_13_ | 0,92900 | -0,77100 | 0,21920 | -0,55180 | -0,22830 | -0,25420 | 0,02910 | 0,02420 |
| *C2/m* | LaH_8_ | 0,88900 | -0,52480 | 0,20630 | -0,31840 | -0,35250 | -0,38190 | 0,01520 | 0,02460 |
| *P-1* | La_3_H_26_ | 0,89700 | -0,57270 | 0,21120 | -0,36150 | -0,32890 | -0,35530 | 0,01810 | 0,03130 |
| *Cmcm* | LaH_9_ | 0,90 | -0,60650 | 0,21910 | -0,38730 | -0,33050 | -0,34970 | 0,01110 | 0,02950 |
| *P6/mmm* | LaH_16_ | 0,94100 | -0,83340 | 0,21400 | -0,61940 | -0,17310 | -0,20700 | 0,03810 | 0,02430 |
| *Pm-3n* | La_4_H_23_ | 0,85200 | -0,26240 | 0,18580 | -0,07660 | -0,43590 | -0,47710 | 0,02510 | 0,01910 |
| *Cmcm* | LaH_3_ | 0,75 | 0,47480 | 0,19560 | 0,67040 | -0,64960 | -0,65710 | 0,00 | 0,00 |
| *R-3m* | La_2_H_5_ | 0,71400 | 0,83010 | 0,18910 | 1,01920 | -0,62760 | -0,63330 | 0,00210 | 0,00820 |
| *I4/mmm* | LaH_4_ | 0,80 | 0,08260 | 0,18800 | 0,27060 | -0,57490 | -0,60180 | 0,00 | 0,00 |
| *P6/mmm* | LaH_2_ | 0,66700 | 1,29180 | 0,17300 | 1,46480 | -0,61050 | -0,62110 | 0,00 | 0,00 |
| *Fm-3m* | La | 0,00 | 8,12600 | 0,02700 | 8,15290 | 0,00 | 0,00 | 0,00 | 0,00 |
| *Cmc2_1* | LaH_7_ | 0,87500 | -0,44290 | 0,23010 | -0,21290 | -0,40030 | -0,40270 | 0,20 | 0,03860 |
| *Immm* | La_3_H_13_ | 0,81200 | -0,00250 | 0,19660 | 0,19410 | -0,54340 | -0,56460 | 0,00670 | 0,01280 |
| *P2_1/c* | H | 1,00 | -1,20950 | 0,26180 | -0,94770 | 0,00 | 0,00 | 0,00 | 0,00 |
| *Im-3m* | LaH_6_ | 0,85700 | -0,30910 | 0,17550 | -0,13360 | -0,43320 | -0,48600 | 0,01630 | 0,00 |
| *Cmmm* | La_3_H_10_ | 0,76900 | 0,34050 | 0,19630 | 0,53690 | -0,60430 | -0,61560 | 0,01310 | 0,02050 |
| *P-1* | LaH_5_ | 0,83300 | -0,15900 | 0,19900 | 0,04 | -0,50540 | -0,52910 | 0,00 | 0,00570 |

**Table S2.** Enthalpies of formation of various lanthanum hydrides (in eV/atom) at 120 GPa and 0 K (Figure S2). ZPE stands for the zero-point energy. X, Y are coordinates of lanthanum hydrides on the two-dimensional convex hull diagram. Fitness is the distance from the convex hull line.

| **Space Group** | **Formula** | **X** | **E** | **ZPE** | **E+ZPE** | **Y** | **ZPE Y** | **Fitness without ZPE** | **ZPE fitness** |
| --- | --- | --- | --- | --- | --- | --- | --- | --- | --- |
| *Fm-3m* | LaH_10_ | 0,90900 | -0,22280 | 0,16470 | -0,05800 | -0,29430 | -0,37440 | 0,02550 | 0,00 |
| *C2/m* | La_2_H_9_ | 0,81800 | 0,54580 | 0,20900 | 0,75480 | -0,53020 | -0,54440 | 0,01120 | 0,01650 |
| *C2/m* | La_3_H_26_ | 0,89700 | -0,13350 | 0,21010 | 0,07660 | -0,34360 | -0,37530 | 0,02230 | 0,02450 |
| *P4/nmm* | LaH_11_ | 0,91700 | -0,32380 | 0,23210 | -0,09160 | -0,31160 | -0,32600 | 0,00 | 0,01550 |
| *C2/m* | LaH_23_ | 0,95800 | -0,62820 | 0,25530 | -0,37290 | -0,15580 | -0,15680 | 0,80 | 0,01600 |
| *P6_3/mmc* | LaH_9_ | 0,90 | -0,16390 | 0,20280 | 0,03900 | -0,33590 | -0,37560 | 0,01610 | 0,01780 |
| *C2/m* | LaH_8_ | 0,88900 | -0,07070 | 0,21110 | 0,14050 | -0,36550 | -0,39430 | 0,01600 | 0,02240 |
| *Immm* | La_3_H_13_ | 0,81200 | 0,59440 | 0,20540 | 0,79980 | -0,54440 | -0,56080 | 0,00730 | 0,01190 |
| *R-3m* | LaH_13_ | 0,92900 | -0,39150 | 0,21570 | -0,17580 | -0,24790 | -0,28150 | 0,02240 | 0,01060 |
| *Pm-3n* | La_4_H_23_ | 0,85200 | 0,26420 | 0,19630 | 0,46050 | -0,43980 | -0,47470 | 0,02830 | 0,01940 |
| *Cmcm* | LaH_3_ | 0,75 | 1,20050 | 0,20370 | 1,40430 | -0,62870 | -0,63210 | 0,00 | 0,00 |
| *R-3m* | La_2_H_5_ | 0,71400 | 1,60400 | 0,19630 | 1,80030 | -0,61980 | -0,62220 | 0,00210 | 0,00440 |
| *I4/mmm* | LaH_4_ | 0,80 | 0,70150 | 0,19800 | 0,89950 | -0,57540 | -0,59630 | 0,00 | 0,00 |
| *P6/mmm* | LaH_2_ | 0,66700 | 2,13760 | 0,18030 | 2,31790 | -0,61240 | -0,61940 | 0,00 | 0,00 |
| *Fm-3m* | La | 0,00 | 10,11560 | 0,02970 | 10,14530 | 0,00 | 0,00 | 0,00 | 0,00 |
| *P6/mmm* | LaH_16_ | 0,94100 | -0,47160 | 0,21550 | -0,25620 | -0,18870 | -0,22560 | 0,02920 | 0,01710 |
| *Cmc2_1* | LaH_7_ | 0,87500 | 0,06060 | 0,23610 | 0,29670 | -0,38760 | -0,38820 | 0,02420 | 0,05810 |
| *Im-3m* | LaH_6_ | 0,85700 | 0,20360 | 0,19 | 0,39360 | -0,44190 | -0,48430 | 0,01430 | 0,00 |
| *Cmmm* | La_3_H_10_ | 0,76900 | 1,01130 | 0,20290 | 1,21420 | -0,60550 | -0,61430 | 0,00120 | 0,00420 |
| *P-1* | LaH_5_ | 0,83300 | 0,39880 | 0,20830 | 0,60710 | -0,50980 | -0,52830 | 0,00 | 0,00320 |
| *C2/c* | H | 1,00 | -0,93280 | 0,26620 | -0,66660 | 0,00 | 0,00 | 0,00 | 0,00 |

**Table S3.** Enthalpies of formation of various lanthanum hydrides (in eV/atom) at 150 GPa and 0 K (Figure S3). ZPE stands for the zero-point energy. X, Y are coordinates of lanthanum hydrides on the two-dimensional convex hull diagram. Fitness is the distance from the convex hull line.

| **Space Group** | **Formula** | **X** | **E** | **ZPE** | **E+ZPE** | **Y** | **ZPE Y** | **Fitness without ZPE** | **ZPE fitness** |
| --- | --- | --- | --- | --- | --- | --- | --- | --- | --- |
| *P-1* | LaH_5_ | 0,83300 | 1,18930 | 0,21930 | 1,40860 | -0,50900 | -0,52140 | 0,00 | 0,01960 |
| *C2/c* | H | 1,00 | -0,55310 | 0,27 | -0,28300 | 0,00 | 0,00 | 0,00 | 0,00 |
| *Fm-3m* | LaH_10_ | 0,90900 | 0,35600 | 0,16370 | 0,51970 | -0,31900 | -0,40440 | 0,01730 | 0,00 |
| *Cmcm* | LaH_4_ | 0,80 | 1,57860 | 0,21760 | 1,79620 | -0,57 | -0,57640 | 0,00450 | 0,02390 |
| *Cmcm* | LaH_3_ | 0,75 | 2,22530 | 0,21300 | 2,43830 | -0,59870 | -0,59820 | 0,00530 | 0,00950 |
| *I4/mmm* | LaH_4_ | 0,80 | 1,57510 | 0,19720 | 1,77230 | -0,57350 | -0,60030 | 0,00 | 0,00 |
| *P6/mmm* | LaH_2_ | 0,66700 | 3,33740 | 0,18570 | 3,52300 | -0,61230 | -0,62 | 0,00 | 0,00 |
| *Fm-3m* | La | 0,00 | 12,95520 | 0,04 | 12,99510 | 0,00 | 0,00 | 0,00 | 0,00 |
| *C2/m* | La_3_H_26_ | 0,89700 | 0,48300 | 0,22040 | 0,70340 | -0,36140 | -0,38720 | 0,01800 | 0,03880 |
| *R-3m* | LaH_13_ | 0,92900 | 0,14100 | 0,22960 | 0,37050 | -0,27080 | -0,29490 | 0,01490 | 0,02060 |
| *P4/nmm* | LaH_11_ | 0,91700 | 0,24290 | 0,24020 | 0,48320 | -0,32970 | -0,34030 | 0,00 | 0,02850 |
| *Pm-3n* | La_4_H_23_ | 0,85200 | 1,01160 | 0,20940 | 1,22100 | -0,43650 | -0,46310 | 0,03480 | 0,04370 |
| *C2/m* | LaH_23_ | 0,95800 | -0,15650 | 0,26540 | 0,10900 | -0,16620 | -0,16120 | 0,40 | 0,02540 |
| *P6_3/mmc* | LaH_9_ | 0,90 | 0,45140 | 0,21310 | 0,66460 | -0,34630 | -0,38020 | 0,02290 | 0,04040 |
| *C2/m* | LaH_8_ | 0,88900 | 0,56940 | 0,22200 | 0,79140 | -0,37850 | -0,40090 | 0,01360 | 0,03940 |
| *P6/mmm* | LaH_16_ | 0,94100 | 0,02690 | 0,22920 | 0,25610 | -0,21460 | -0,24200 | 0,01910 | 0,02020 |
| *C2/m* | LaH_7_ | 0,87500 | 0,74220 | 0,20890 | 0,95110 | -0,39330 | -0,42560 | 0,02710 | 0,03990 |
| *R-3m* | LaH_6_ | 0,85700 | 0,92860 | 0,21260 | 1,14120 | -0,44810 | -0,47260 | 0,01130 | 0,02530 |
| *Cmmm* | La_3_H_10_ | 0,76900 | 1,96300 | 0,21470 | 2,17760 | -0,60130 | -0,60350 | 0,00 | 0,00140 |

**2. Electron, phonon, elastic and superconducting properties of La_4_H_23_**


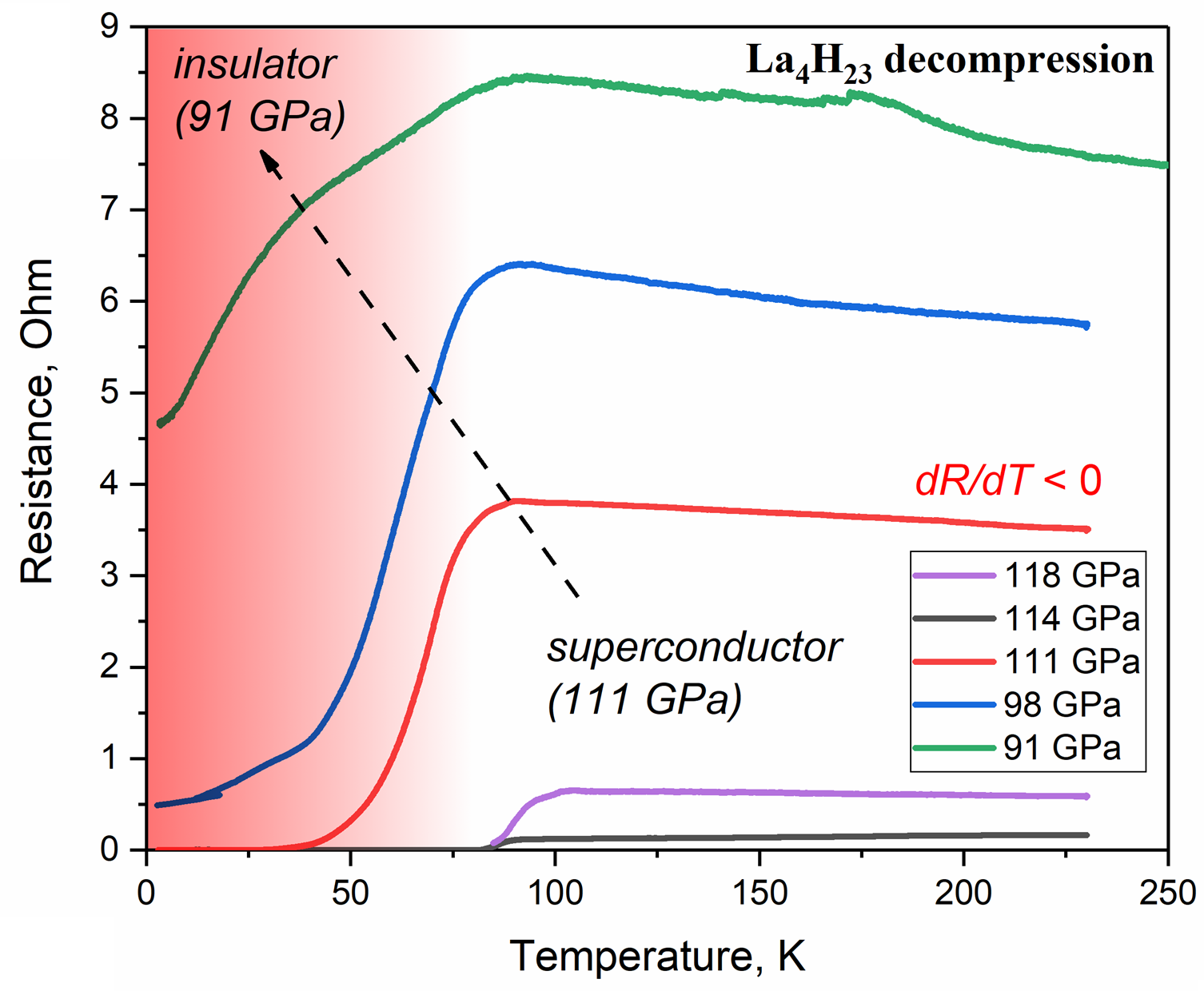


**Figure S7.** Experimental transport properties of La_4_H_23_ in DAC H1. (a) Decompression of the DAC H1 and temperature dependence of the electrical resistivity of the sample in real scale. Pronounced increase in electrical resistance in 10-100 times at *T* > *T*_C_ is observed during decompression due to the beginning of the metal-insulator transition. The anomaly at 114 GPa is an exception. (b) Residual resistance of the sample detected in the SC state in magnetic fields of 0-4 T.


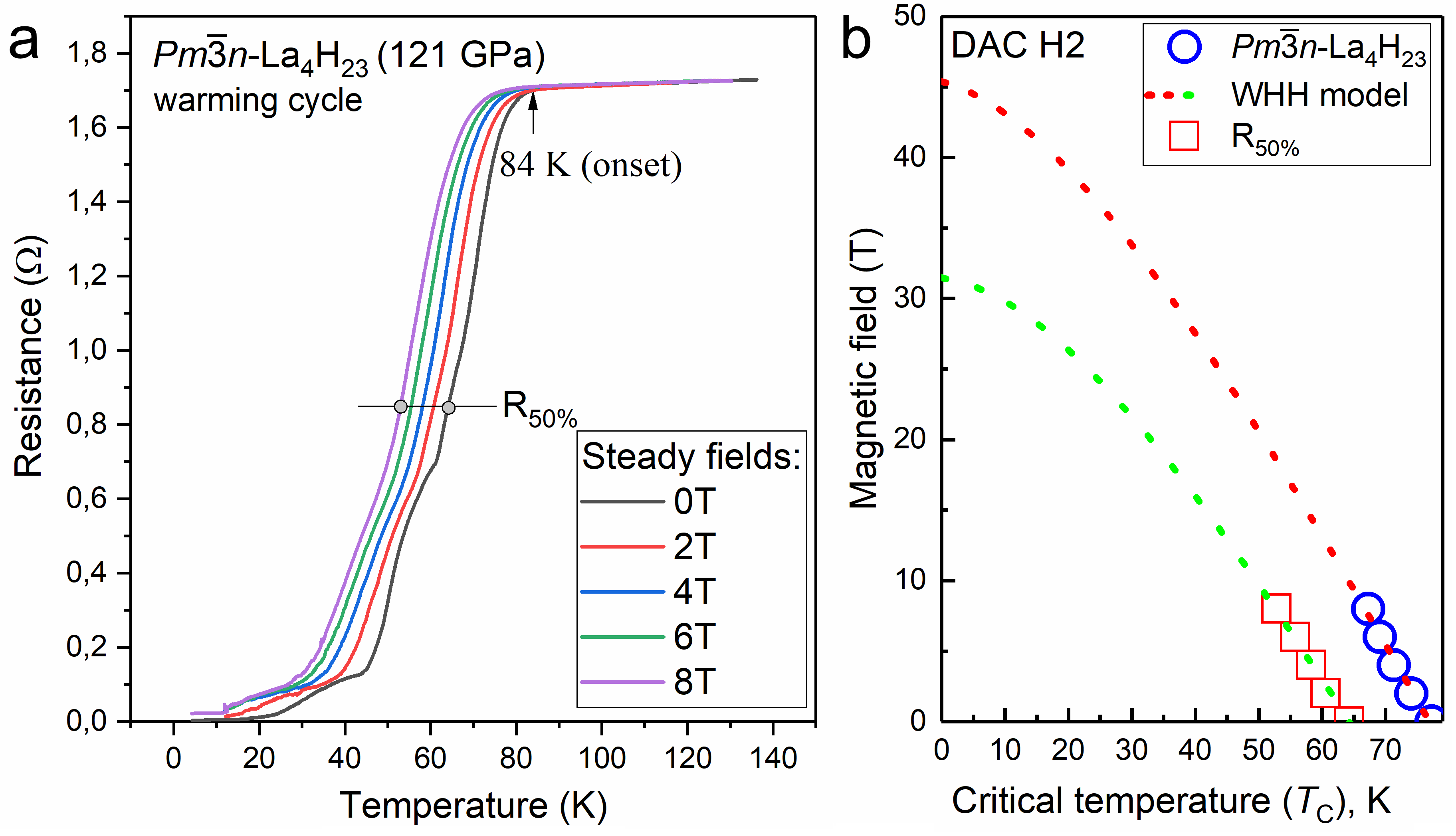


**Figure S8.** Experimental transport properties of La_4_H_23_ in DAC H2 before the pulsed field study. (a) Temperature dependence of electrical resistance of La_4_H_23_ at 121 GPa measured in magnetic fields 0-8 T in warming cycles. (b) Upper critical magnetic field *μ_0_H_C2_(T)* dependence on temperature reconstructed using onset *T*_C_ and *T*_C_ found via R_50%_ criteria. Extrapolations based on the WHH model^[1]^ are shown by red and green dashed lines.


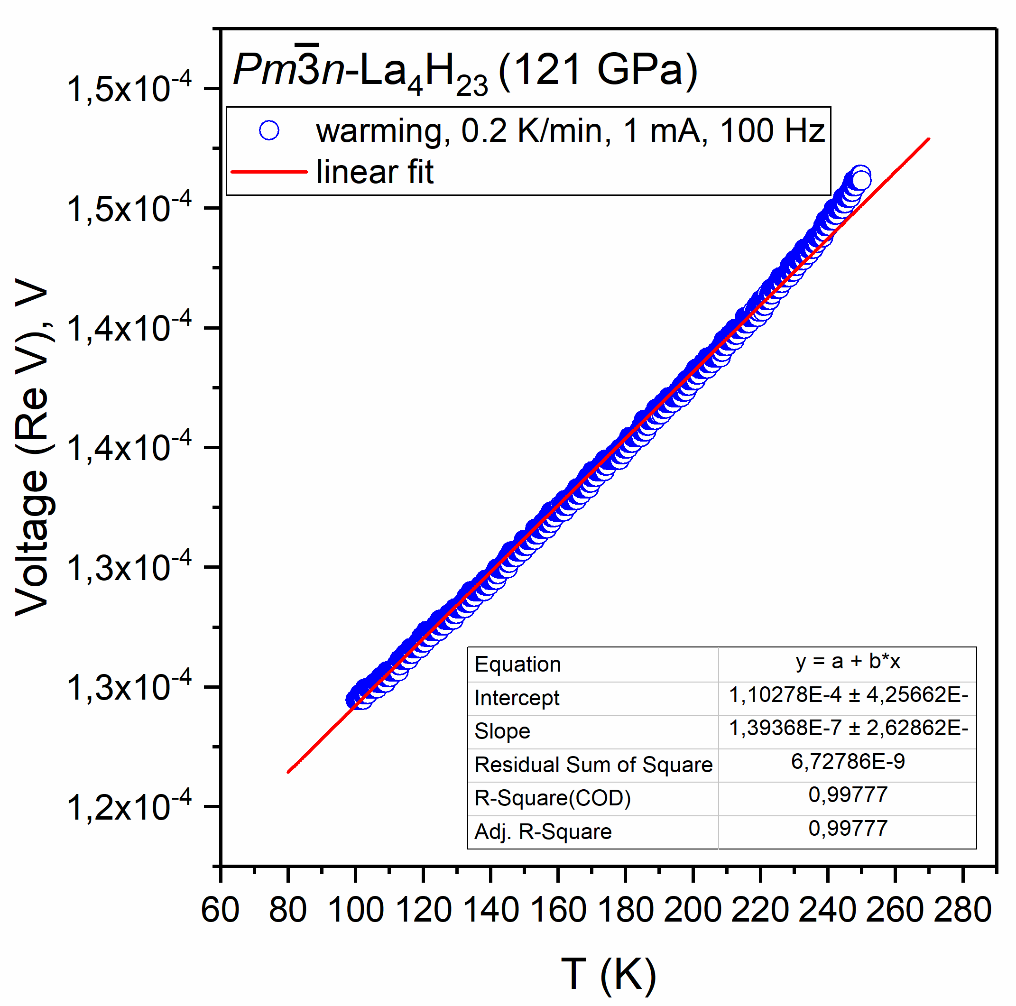


**Figure S9.** Quasi *T*-linear dependence of the voltage drop across the DAC H2 sample during 4-contact measurements in AC mode on temperature in the range from 100 K to 260 K. In fact, this *R(T)* curve cannot be explained within the framework of the electron-phonon scattering model (e.g., Bloch-Grüneisen formula) and indicates the non-Fermi liquid behavior of the sample.


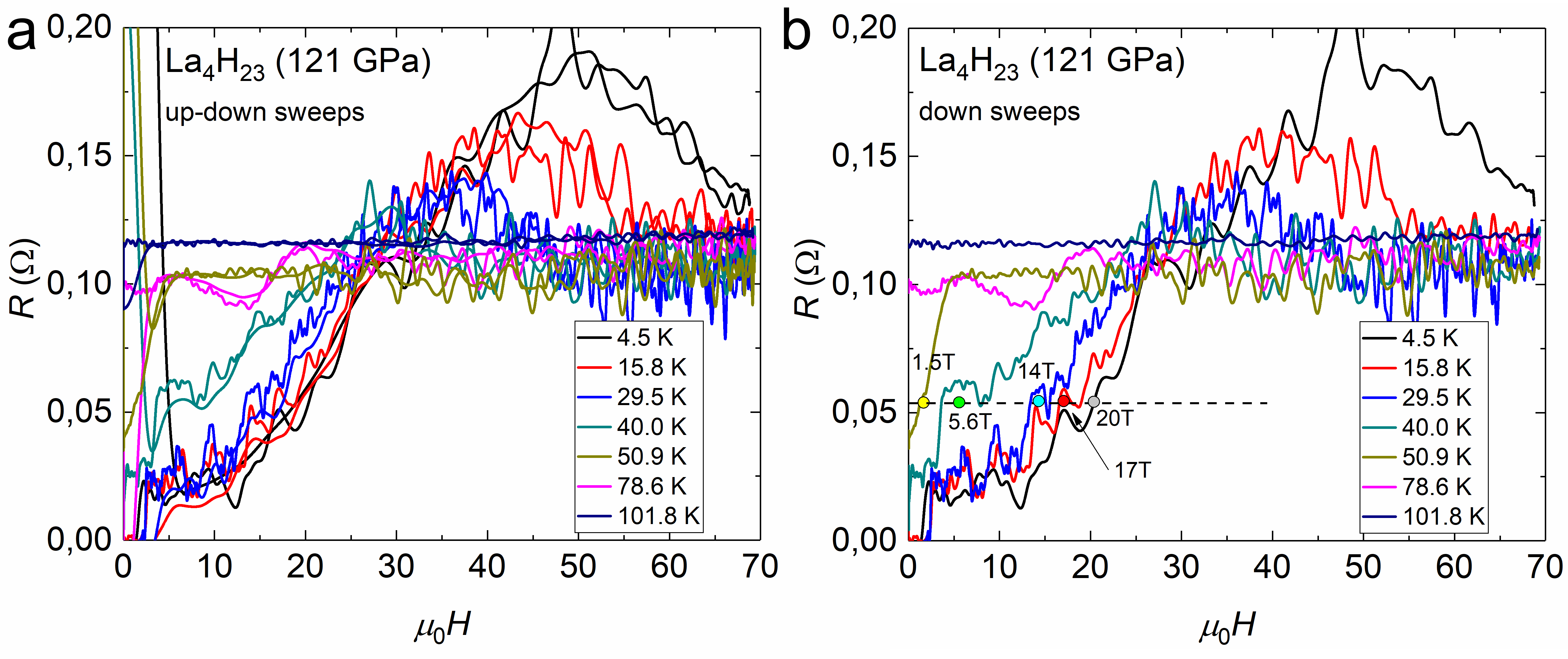


**Figure S10.** Dependence of the electrical resistance of the sample in DAC H2 on the external magnetic field *R(H)* measured in the AC mode at frequencies of 16.66 kHz (4.5 K) and 33.33 kHz (most cases). Raw data. Such loud periodic noise is caused by large open loops in the contact wires of the DAC H2. (a) Magnetic field sweeps up and down together; (b) only sweeps down are shown and used to determine *μ_0_H_C2_(T)* according to *R_50%_* criteria.


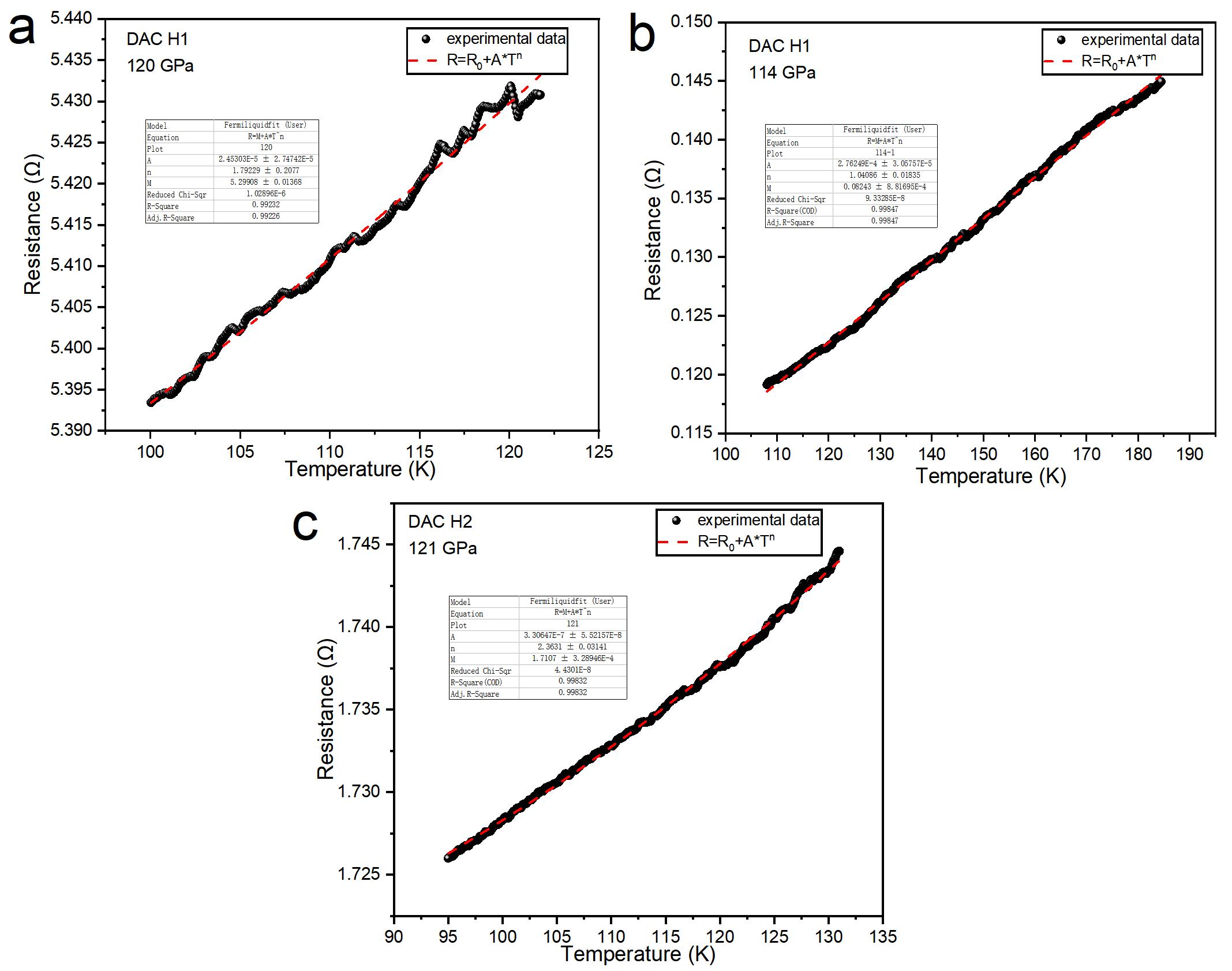


**Figure S11**. The resistance dependence of temperature on the non-superconducting state of La_4_H_23_. We used the target fitting form: *R(T)=M+AT^n^*. (a) DAC H1 at 120 GPa in the temperature range from 100 K to 120 K, *n***≈**1.8. (b) Interpolation at *T*>110 K, *n***≈**1. (c) DAC H2 at 121 GPa, *T*>95 K, *n***≈**2.4.


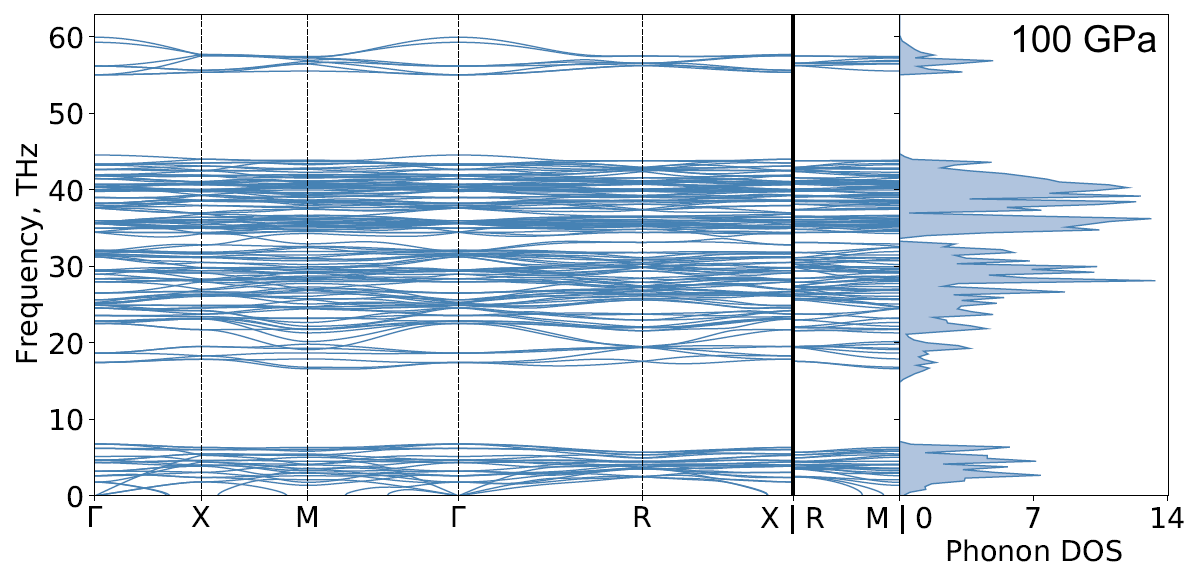


**Figure S12**. Phonon band structure and corresponding density of states of La_4_H_23_ at 100 GPa in harmonic approximation. The compound has a small number of imaginary phonon modes, which are likely to disappear when anharmonic effects are considered.

**
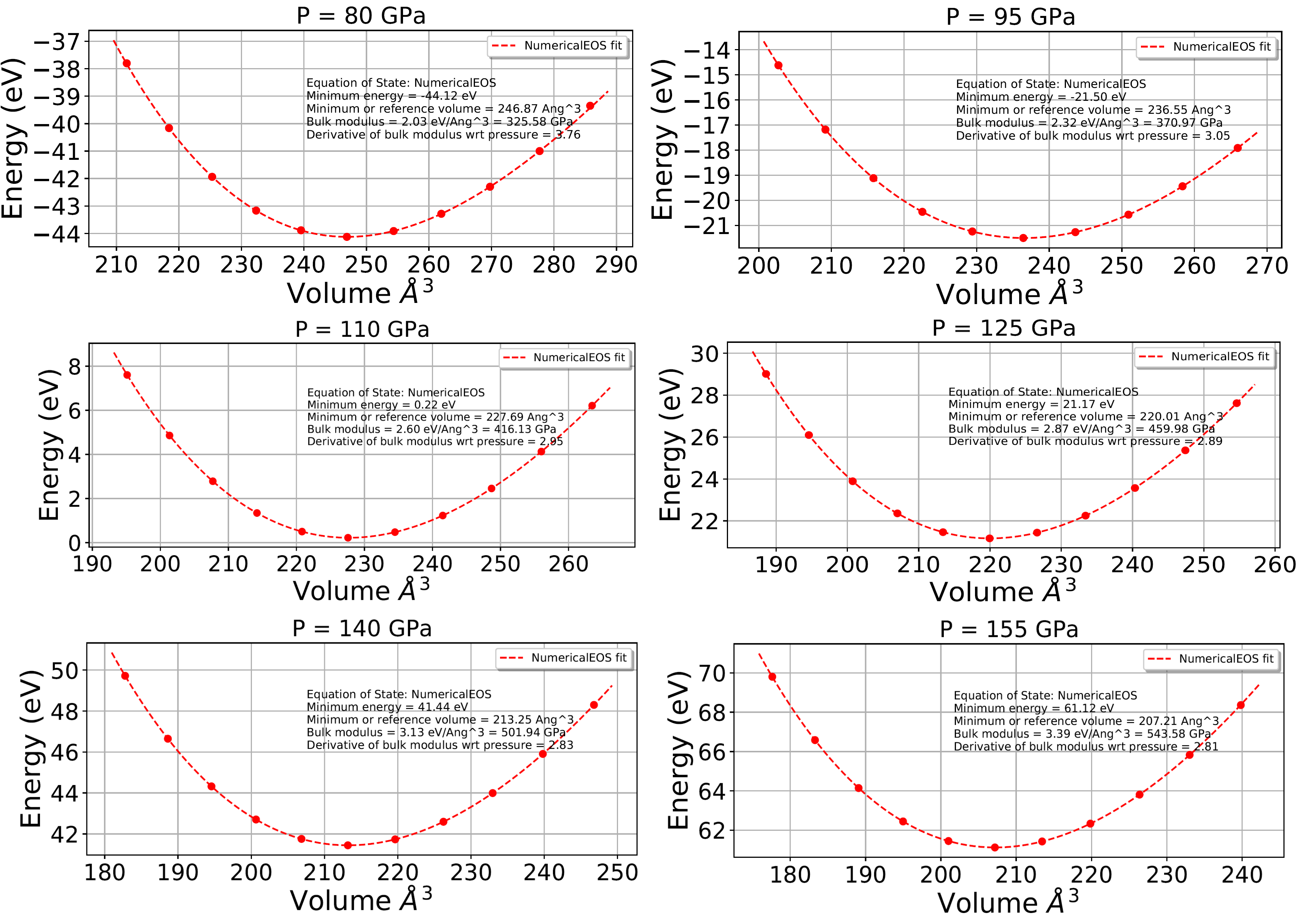
**

**Figure S13**. Equation of state, bulk modulus (*B*) and its derivative (*dB/dP*) of La_4_H_23_ at pressures from 80 to 155 GPa. Calculations were done using VASP code with the PAW PBE pseudopotentials for La and H.

t

**Figure S14.** Superconducting state parameters and Eliashberg function for La_4_H_23_ (54 atoms in the unit cell) at 150 GPa calculated using PBE HGH pseudopotentials for La and H atoms, *q*-mesh was 2×2×2. Pictures were generated via python script available on GitHub^[2]^.

**REFERENCES**

[1] Werthamer NR, Helfand E, Hohenberg PC. Temperature and purity dependence of the superconducting critical field,hc2. Iii. Electron spin and spin-orbit effects. *Phys Rev*, 1966, 147: 295-302

[2] A2f - superconducting properties calculation. <https://githubcom/GitGreg228/a2f>, 2023,
